# Proprioceptive Bimanual Test in Intrinsic and Extrinsic Coordinates

**DOI:** 10.3389/fnhum.2015.00072

**Published:** 2015-02-18

**Authors:** Riccardo Iandolo, Valentina Squeri, Dalia De Santis, Psiche Giannoni, Pietro Morasso, Maura Casadio

**Affiliations:** ^1^NeuroLab, Department of Informatics, Bioengineering, Robotics and Systems Engineering, University of Genoa, Genoa, Italy; ^2^Motor Learning and Robotic Rehabilitation Laboratory, Department of Robotics, Brain and Cognitive Sciences, Istituto Italiano di Tecnologia, Genoa, Italy; ^3^ART Education and Rehabilitation Center, Genoa, Italy

**Keywords:** proprioception, intrinsic coordinates, extrinsic coordinates, robotics, bimanual task, position sense

## Abstract

Is there any difference between matching the position of the hands by asking the subjects to move them to the same spatial location or to mirror-symmetric locations with respect to the body midline? If the motion of the hands were planned in the extrinsic space, the mirror-symmetric task would imply an additional challenge, because we would need to flip the coordinates of the target on the other side of the workspace. Conversely, if the planning were done in intrinsic coordinates, in order to move both hands to the same spot in the workspace, we should compute different joint angles for each arm. Even if both representations were available to the subjects, the two tasks might lead to different results, providing some cue on the organization of the “body schema”. In order to answer such questions, the middle fingertip of the non-dominant hand of a population of healthy subjects was passively moved by a manipulandum to 20 different target locations. Subjects matched these positions with the middle fingertip of their dominant hand. For most subjects, the matching accuracy was higher in the extrinsic modality both in terms of systematic error and variability, even for the target locations in which the configuration of the arms was the same for both modalities. This suggests that the matching performance of the subjects could be determined not only by proprioceptive information but also by the cognitive representation of the task: expressing the goal as reaching for the physical location of the hand in space is apparently more effective than requiring to match the proprioceptive representation of joint angles.

## Introduction

Proprioception is defined as the ability to sense body position and movement in the absence of visual guidance (Sherrington, 1907; Dickinson, 1976; Brookhart et al., 1984). The primary source of proprioceptive information is the muscle spindles (Winter et al., 2005; Proske and Gandevia, 2009) and cutaneous and joint receptors (Collins et al., 2005; Proske and Gandevia, 2012). Afferent information from these receptors is processed in cortical regions including primary and secondary sensorimotor areas and subcortical regions including the basal ganglia and the cerebellum (Naito et al., 2002; Hagura et al., 2009; Boisgontier and Swinnen, 2014). Moreover, Proske and Gandevia (2012) showed that proprioception is modulated by central descending signals and a number of studies (Ansems et al., 2006; Gandevia et al., 2006; Walsh et al., 2013) demonstrated that motor commands contribute to the joint position sense.

Proprioceptive feedback plays a crucial role in daily life and in the interactions with the world around us. Since position sense contributes to the control of posture and motion, proprioceptive deficits may compromise the ability to perform everyday activities and may interfere with motor learning processes as well as with the recovery after stroke (Kusoffsky et al., 1982; Rand et al., 1999; Schabrun and Hillier, 2009). For these reasons, it is important not only to quantify position sense but also to understand the underlying mechanisms, and how the cortical areas process proprioceptive feedback. Unfortunately, clinical assessment of proprioceptive deficits still lacks the necessary reliability and accuracy to discriminate sensorimotor impairments and therefore to plan specific therapeutic interventions.

In order to fill this gap, a number of research groups developed methods based on robotics (Carey et al., 1996; Wilson et al., 2010; Squeri et al., 2011; Dukelow et al., 2012; Semrau et al., 2013), optoelectronics (Schmidt et al., 2013), and magnetic devices (Leibowitz et al., 2008) and different testing protocols were proposed (see Goble, 2010 for a review).

In the present work, we focus on “contralateral concurrent matching tasks,” i.e., bimanual tasks where one hand is positioned at a location and the subjects match this position with the other hand.

In contrast to unilateral matching tasks, bimanual matching tasks have been shown to be more challenging to execute and healthy subjects present different matching errors (ME) when evaluated with bilateral and unilateral tests (Adamo and Martin, 2009; Goble and Brown, 2009). Moreover, bimanual tasks are deemed to account for asymmetry between the two hands (Adamo and Martin, 2009; Martin and Adamo, 2011; Adamo et al., 2012): the dominant hand may use preferentially feedforward control (Bagesteiro and Sainburg, 2002; Wang and Sainburg, 2007; Przybyla et al., 2013) and be more accurate when matching targets with a predominant visual nature (Goble and Brown, 2008) whereas the other hand is likely to use preferentially feedback control and be more accurate when matching targets, with a predominant proprioceptive nature (Goble and Brown, 2008). However, the difference between feedforward and feedback control is probably a limited line of explanation: asymmetry may also depend on other factors such as the relative gains of the different sensorimotor systems (Adamo and Martin, 2009; Plaisier and Ernst, 2012; Squeri et al., 2012; Wong et al., 2014), attentional bias (Serrien, 2009; Buckingham et al., 2010), and responsibility assignment (White and Diedrichsen, 2010).

Bimanual tests can be performed either in extrinsic – hand space – coordinates (Leibowitz et al., 2008) or in intrinsic – joints space – coordinates (Goble, 2010; Semrau et al., 2013). Several studies investigated proprioception, mostly in unilateral tasks, by studying either the fingertip/hand position in the workspace (i.e., by using extrinsic-coordinate tests: (Crowe et al., 1987; van Beers et al., 1996, 1998) or the elbow angle in the joint space (i.e., by using intrinsic-coordinate tests: (Soechting, 1982; Darling, 1991; Zia et al., 2002; Gritsenko et al., 2007). Recently, Fuentes and Bastian (2010) compared the performance of subjects in a unilateral task when asked to match a pointer either to elbow angles or to fingertip positions. These authors found greater accuracy for fingertip matching than elbow angle matching suggesting that “the brain has better access to limb endpoint position than joint angles.” This is in agreement with the growing evidence (Kalaska et al., 1990; Prud’homme and Kalaska, 1994; Tillery et al., 1996) that the CNS represents limb locations in the workspace as endpoint positions.

This observation suggests that the same difference may be present in bimanual proprioceptive tasks when subjects match the position of their fingertips or the position of their joints, so that the two conditions may lead to different position estimates. Here, we aim at evaluating in a quantitative term the difference, if present, in repeatability, accuracy, and space representation when executing bimanual proprioceptive tests in extrinsic or intrinsic coordinates. Thus, we asked a group of healthy right-handed (or ambidextrous) subjects to match the position of their left middle fingertip in two alternative ways: by moving the right one either in the same location in the workspace (extrinsic coordinates) or in mirror-symmetric locations with respect to their body midline (intrinsic coordinates). In addition, we investigated how the position sense maps across the two-dimensional space of the task under these two different testing conditions.

## Materials and Methods

### Subjects

Twenty-three healthy subjects (age: 29.30 ± 7.08 SD years, 10 males) with no history of neurological or musculoskeletal disorders participated in the present study. They were recruited among the students and employees of the University of Genoa. Their handedness was assessed by two inventories: the Edinburgh inventory (Oldfield, 1971) and Dutch test (van Strien, 1992). According to these evaluations, two subjects were ambidextrous while all the others were right-handed (Dutch: 8.12 ± 2.46 SD, Edinburgh: 76.41 ± 24.27 SD). This research study conforms to the standard of the declaration of Helsinki and was approved by the institutional ethical committee. All subjects provided written informed consent prior to participation in the study. The experiments were carried out at the NeuroLab, Department of Informatics, Bioengineering, Robotics and Systems Engineering of the University of Genoa (Genoa, Italy).

### Experimental set-up

**Figure 1:**
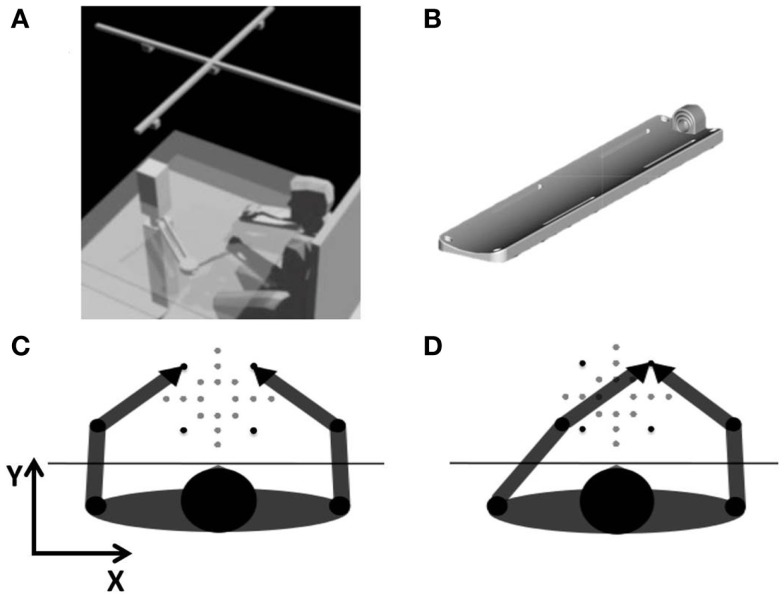
**(A)** Experimental set-up. A planar manipulandum passively moved the left arm in different workspace locations. Subjects matched the position of the middle fingertips by actively moving the right arm. An array of four infrared cameras was mounted on the ceiling. **(B)** Arm support with different-sized middle fingertip holder. **(C)** Intrinsic-matching test: test in intrinsic coordinates. Goal: the two middle fingertips in mirror-symmetric locations of the workspace. **(D)** Extrinsic-matching test: test in extrinsic coordinates. Goal: the two middle fingertips in the same workspace location. The black targets represent the vertices of the 14 cm × 14 cm square and were used to compute variability.

The subjects sat blindfolded in front of a desk, with the center of the workspace aligned with the sagittal midline of their body (Figure 1A). A chair provided a secure back support and two belts prevented appreciable trunk movements while allowing shoulders and elbow rotations. The height of the chair was adjusted to position the table at the chest level. Chair and table did not move during the experiment. Subjects’ forearms (from the elbow to the middle finger tip) were restrained palm down on two identical custom-built supports with all the fingers extended. Subjects’ tips of both middle fingers were encapsulated in a plastic holder, adjustable with respect to the fingers sizes of the subject (Figure 1B). The left forearm support was linked to the end-effector of a planar manipulandum (Casadio et al., 2006) and positioned slightly below the table surface. The link was implemented by a non-actuated, low-friction rotational joint, which connected the arm support and the robot’s end-effector in correspondence of the tip of the middle finger. The right forearm support could freely slide on top of the table surface, thanks to carefully built, low-friction contacts. The two holders lay on two horizontal planes vertically separated by 10 cm, the least possible distance for avoiding interference between the two arms in all subjects within our experimental set-up.

The position of the left middle fingertip was recorded by using the manipulandum encoders, whereas the position of the right middle fingertip was measured optically. An array of four infrared cameras (V120 slim, NaturalPoint Inc., OR, USA: software: C++ custom modification of NaturalPoint SDK) located 2 m above the table recorded the position of three infrared active markers: one aligned with the right hand fingertip and the other with the two acromions. The latter markers were also used to verify the absence of significant trunk movements. The robot and the cameras were synchronized and the entire set-up was calibrated. The positions of both fingertips were sampled at 60 Hz and the corresponding reconstruction error on the entire workspace was <2 mm.

### Proprioceptive bimanual task

The manipulandum passively moved the subjects’ left hand to 20 different target positions presented in random order over a 21 cm × 21 cm workspace, centered with respect to the subjects’ body midline (Figures 1C,D). Participants were required to actively move their right hand in order to match the left hand according to two different matching modalities:

- Extrinsic-matching test: the tips of the two middle fingers should be coincident and centered in the current target position;
- Intrinsic-matching test: the tip of the right middle finger should duplicate the position of the left fingertip in a mirror-symmetric way, with respect to the body midline.

In other words, the task was designed such that at the end of each movement set, the two hands had reached the same positions of the task space in the corresponding modality.

The assessment protocol was articulated in four movement sets, of 40 trials each. A trial consisted of the following steps, starting from a standard position for both hands:

– Subjects were instructed to let the robotic device move passively their left hand, while maintaining the initial position of the right hand. In particular, starting from a target position ${\vec{x}}_{i}$, the manipulandum carried the left arm to another target ${{\vec{x}}_{i}}_{+1}$, following a linear, minimum-jerk profile:
  (1) $${\vec{x}}_{i+1}={\vec{x}}_{i}+\left({\vec{x}}_{i+1}-{\vec{x}}_{i}\right)\left[6\xi^{5}-15\xi^{4}+10\xi^{3}\right]\;\mathrm{with}\;\xi =t∕T\left(T=1s\right)$$
– As soon as the manipulandum brought the left hand in the target position, an acoustic prompt was delivered. The subject was instructed to start moving the other hand, according to the predefined matching modality, and to confirm verbally when he/she believed to have completed the matching operation. It is important to note that there was no time or path constraint in executing the matching task.
– In this phase, subjects were instructed to hold this position until a new target and a new prompt sound were presented. The position of both hands was recorded for 2 s before the operator started a new trial: the robot moved passively the left hand to the next target and the procedure was iterated until the completion of the trial.

The experimental protocol included four movement sets (Figures 1C,D). In each movement set, 40 targets were presented, lying on the vertices of five squares, for a total of 20 different target positions; the vertices of the 14 cm × 14 cm square were repeated at least four times in each movements set to compute the matching inter-trial variability. In order to verify to which extent the predictability of the target sequence could affect the results, we alternated two different presentation modalities:

- Random: targets were presented in completely random order.
- Sequence of squares: targets were presented in quadruplets of ordered targets, each quadruplet corresponding to a sequence of adjacent vertices of a square. The order of presentation of these four target blocks during a movement set was random.

The four movement sets corresponded to the different matching modalities: two sets were executed in intrinsic and two in extrinsic coordinates. For each matching modality, one movement set was executed following the random presentation and the other using the sequence of squares presentation. The order of the four movement sets was randomized across subjects while the order of presentation of the targets for each test was the same for all subjects. A session lasted about 45 min.

### Data analysis

#### Upper limb position sense: quantitative assessment

In order to characterize the position sense measured using the two tasks in different coordinate systems, the following indicators, similar to those defined by Dukelow et al. (2010), were computed:

– *Systematic shift*: it quantifies a systematic error between the active and the passive hand. For each target, we computed the *x* and *y* signed components of the distance between the two fingertips and we averaged these signed measures across all targets, obtaining shift_*x*_ and shift_*y*_. Then, we combined them as follows:
  (2) $$\text{Systematic shift}=\sqrt{{\mathrm{shift}}_{x}^{2}+{\mathrm{shift}}_{y}^{2}}$$
  Therefore, the shift measure is different from 0 only if there is a systematic error in the workspace. Note that for computing the measure in the intrinsic test, the *x*-coordinates of the targets were inverted to obtain the mirror-symmetric positions to match.
– *Variability*: it measures the trial-by-trial repeatability of the active hand matching. It was computed on the target positions presented at least four times in each movement set (16 times in total), i.e., the vertices of a 14 cm × 14 cm square (black targets in Figures 1C,D). For each target, the standard deviation (SD) of the *x* and *y*-coordinates of the active moving finger was computed and then averaged across the target set, obtaining var_*x*_ and var_y_. From this, the *variability* index was derived as follows:
  (3) $$\mathrm{Variability}=\sqrt{{\mathrm{var}}_{x}^{2}+{\mathrm{var}}_{y}^{2}}$$
– *Spatial contraction–expansion*: it describes the area of the workspace matched by the active hand relative to one of the passive hand. This parameter examines the spatial distortion of the perceived workspace. Since the 20 targets presented in each movement set were distributed as vertices of concentric squares, the spatial contraction/expansion indicator was computed as the area of each reproduced square, normalized by the area of the squares defined by the target positions. The index was then computed averaging the measures obtained for all squares. Values above 1 represent an expansion of the executed movement and values below 1 correspond to a contraction. However, subjects might perceive the space differently deformed. For having a measure of a systematic spatial deformation error that is independent from the direction of the deformation, the signed error values over all measures obtained for each subject were averaged and then the absolute error was computed when averaging between subjects. This indicator provides information of the systematic spatial error deformation without accounting for its direction, i.e., contraction or expansion were considered equal errors. If the covered areas were equal to those executed by the passive hand, then the spatial contraction–expansion index would be null.

The evaluations of the indicators defined above were performed on the data recorded during the intervals of 2 s in which the robot kept the left hand in the target position and the subject kept the right arm in the matching position, i.e., the same or mirror location with respect to the target, respectively. The positions of both fingertips were averaged across that 2 s.

#### Mapping the accuracy of the position sense across the 2D workspace

In order to evaluate the accuracy of the position sense across the workspace, the matching error (ME) for each target was computed as the Euclidean distance between the two middle fingertips. In particular, the spatial distribution of this error was analyzed, namely its changes in the medio-lateral direction (the left vs. the right side of the body) and in the proximal–distal direction (closer vs. farther positions with respect to the body). Moreover, in order to check if the observed mismatch was due to systematic errors, the same analysis was performed for the signed *x* (MEx) and *y* (MEy) components of the ME. The former component represents a leftward–rightward displacement, while the latter relates to an upward–downward displacement with respect to the target position. Since these are signed measures, if there were no systematic shifts with respect to the target positions, their value would be zero. In summary, in order to fully understand the nature of the performed ME, the signed error measure was computed for each participant and the absolute measure was used only when averaging across participants in order to weigh in the same manner subjects that committed systematic errors in opposite directions.

#### Analysis of trajectory parameters

Although no spatial or temporal constraint was imposed to the subjects during the matching operation, the following indicators characterizing the movement trajectories were evaluated:

– *Reaction time*: time employed by a subject to start moving after the acoustic prompt. The movement onset was defined as the first instant in which the hand speed exceeds 5% of its peak value.
– *Normalized movement duration*: time elapsed between movement onset and the end of the movement (criterion: hand speed >5% of the peak speed) normalized by the duration of the correspondent passive movement imposed by the robot.
– *Number of Peaks*: the peaks in the speed profile were identified taking into account the following two criteria: (1) speed greater than a threshold of 0.02 m/s, (2) temporal distance between adjacent peaks greater than 0.25 s. (This is a measure of movement smoothness and/or the number movements corrections made by the subjects.)

The trajectories were filtered by means of a sixth order Savitzky–Golay filter with a cut-off frequency of ~11 Hz.

#### Statistical analysis

For three indicators – variability, shift, and contraction/expansion, defined as overall measures over the whole workspace – a two-way factorial ANOVA with task modality and order of target presentation as factors was performed. Each factor had two levels: matching test in intrinsic vs. extrinsic coordinates (modality factor) and sequence of squares vs. random sequence (target presentation factor) – ANOVA 2 × 2. The chosen indicators were independent of target location, namely there was an unique measure for each condition that accounts for the performance on all the targets. For the other indicators – ME and its components MEx, MEy – to better understand the dependency of the matching performance on the different target positions in the workspace, a three-way factorial ANOVA was performed by adding as third factor the target location (20 targets) – ANOVA 2 × 2 × 20.

In order to evaluate the relationship between the distance from the body in the forward direction and the ME, a planned comparison was carried out between the targets positioned far from the body (with a *y*-coordinate equal to 10.5 or 7 cm) and close to the body (with a *y*-coordinate equal to −10.5 or −7 cm). The influence of the relative position of the targets in the medio-lateral direction, with respect to the body midline, was also examined. In this case, the planned comparison was performed between targets located rightward (with an *x*-coordinate equal to 10.5 and 7 cm) and leftward (with an *x*-coordinate equal to −10.5 and −7 cm) with respect to the body midline.

For the trajectory parameters defined above – reaction time, movement duration, and number of peaks – a three-way factorial ANOVA was performed in order to analyze their dependency on task modality, order of target presentation, and target position in the workspace – ANOVA 2 × 2 × 20.

## Results

**Figure 2:**
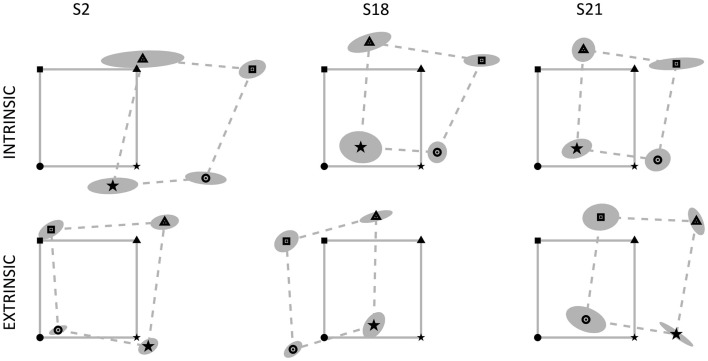
**Data from three subjects with relevant errors in the position matching tasks: S2, S18, and S21**. The continuous gray lines connect the four targets (left fingertip positions) located on the vertices of the 14 cm × 14 cm square, identified by four markers (square, triangle, star, circle). The dotted lines connect the corresponding matching positions achieved by the subjects, surrounded by the uncertainty ellipse. Left and right fingertip positions are identified by the same marker. Top row: intrinsic-matching test; bottom row: extrinsic-matching test.

Subjects actively matched with their right middle fingertip the position of the correspondent left fingertip that was passively moved in different positions of the workspace. We investigated differences in executing the task according to two different modalities, namely reaching (i) the same spatial location or (ii) the mirror-symmetric location with respect to the body midline. Since the ambidextrous subjects exhibited no difference in their performance with respect to the right-handed subjects, we did not consider them separately. In general, subjects adopted different strategies to solve the tasks and we found that each test modality may apparently lead to different and sometimes conflicting conclusions. Let us take, as an example, the behavior of three subjects that made large MEs (S2, S18, and S21; see Figure 2) but, nevertheless, showed a similar proprioceptive bias in the mirror-symmetric test: all of them positioned their right arm rightward with respect to their nominal target location, with S2 exhibiting the greatest errors. In contrast, if we consider the performance of the same subjects in the extrinsic coordinate test, the position errors are greatly different and S2 appears to be the best performer. Therefore, we investigated the following quantitative indicators to better understand the difference of the results obtained under the two different task requirements and their implications in the estimation of the upper limb position sense and its mapping across the workspace.

### Upper limb position sense: Quantitative assessment

#### Variability

**Figure 3:**
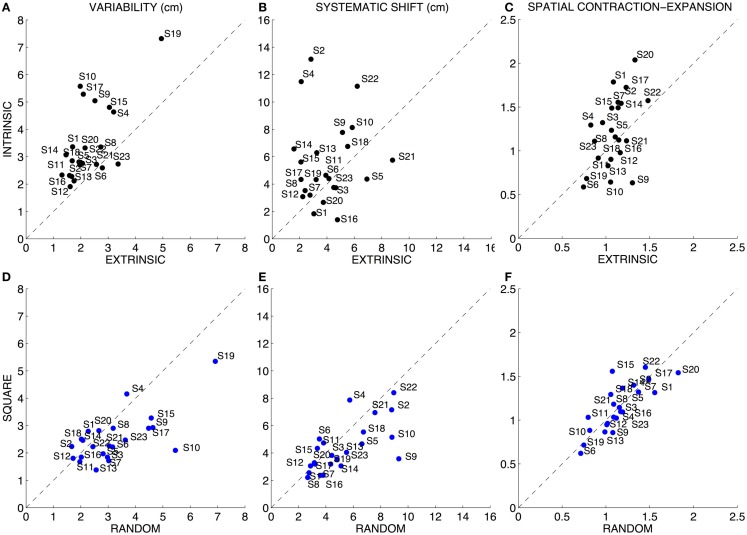
**Quantitative assessment of the position sense**. Left column **(A,D)**: variability (both axes are in centimeters). Central column **(B,E)**: systematic shift (both axes are in centimeters). Right column **(C,F)**: spatial contraction–expansion (both axes are normalized, adimensional). Top row: comparison between the Intrinsic and Extrinsic-matching modality. Bottom row: comparison between the two sequences of target presentation (random vs. sequence of squares). In all the graphs, the dotted line indicates equal performance in the two compared conditions Brookhart et al. (1984).

Subjects exhibited a greater variability [*F*(1,88) = 18.047 *p* < 0.0001] when executing the task in intrinsic coordinates (3.42 ± 1.36 SD cm) with respect to the task in extrinsic coordinates (2.27 ± 0.82 SD cm) (Figures 3A,D). Moreover, the order of target presentation had a significant [*F*(1,88) = 5.897 *p* = 0.017] influence on the trial-by-trial repeatability of the task: subjects were more variable when the targets were presented in random order (3.17 ± 1.30 SD cm) with respect to the movement sets in which the targets were presented in sequence of squares (2.51 ± 0.87 SD cm). Interactions between the two factors (*p* > 0.05) i.e., task modality and order of targets presentation were not significant.

#### Systematic shift

Subjects adopted different strategies; however, most of them [*F*(1,88) = 7.368 *p* = 0.008] had a greater systematic shift when executing the task in intrinsic coordinates (5.56 ± 3.07 SD cm) with respect to the task in extrinsic coordinates (3.99 ± 1.81 SD cm) (Figures 3B,E). There was no significant effect of the order of targets’ presentation (*p* > 0.05) and no significant interaction between the two factors (*p* > 0.05).

#### Spatial contraction–expansion

Subjects exhibited different strategies and we found no clear evidence of a systematic contraction or expansion of the workspace perception for the entire population of subjects, since neither the main tested factors nor their interaction reached significance (Figures 3C,F). This suggests that there is no unique perception of the workspace, but subjects may perceive the space as differently deformed, i.e., contracted or expanded, and their perception may be dependent on the test modality Indeed, the error measure that accounts for the systematic spatial deformation, whether contraction or expansion, showed greater errors [*F*(1,88) = 17.920 *p* < 0.0001] in the movements sets executed in the intrinsic (0.37 ± 0.25 SD) than in the extrinsic test modality (0.17 ± 0.10 SD).

### Map of proprioception across the 2D workspace

#### Matching error

The overall ME changed across the workspace [*F*(19,1760) = 2.7360, *p* < 0.0001] and, as expected, was significantly greater for the intrinsic-matching condition [*F*(1,1760) = 190.55, *p* < 0.0001] and when the targets were presented in random order [*F*(1,1760) = 26.389, *p* < 0.0001]. Interactions were not significant.

**Figure 4:**
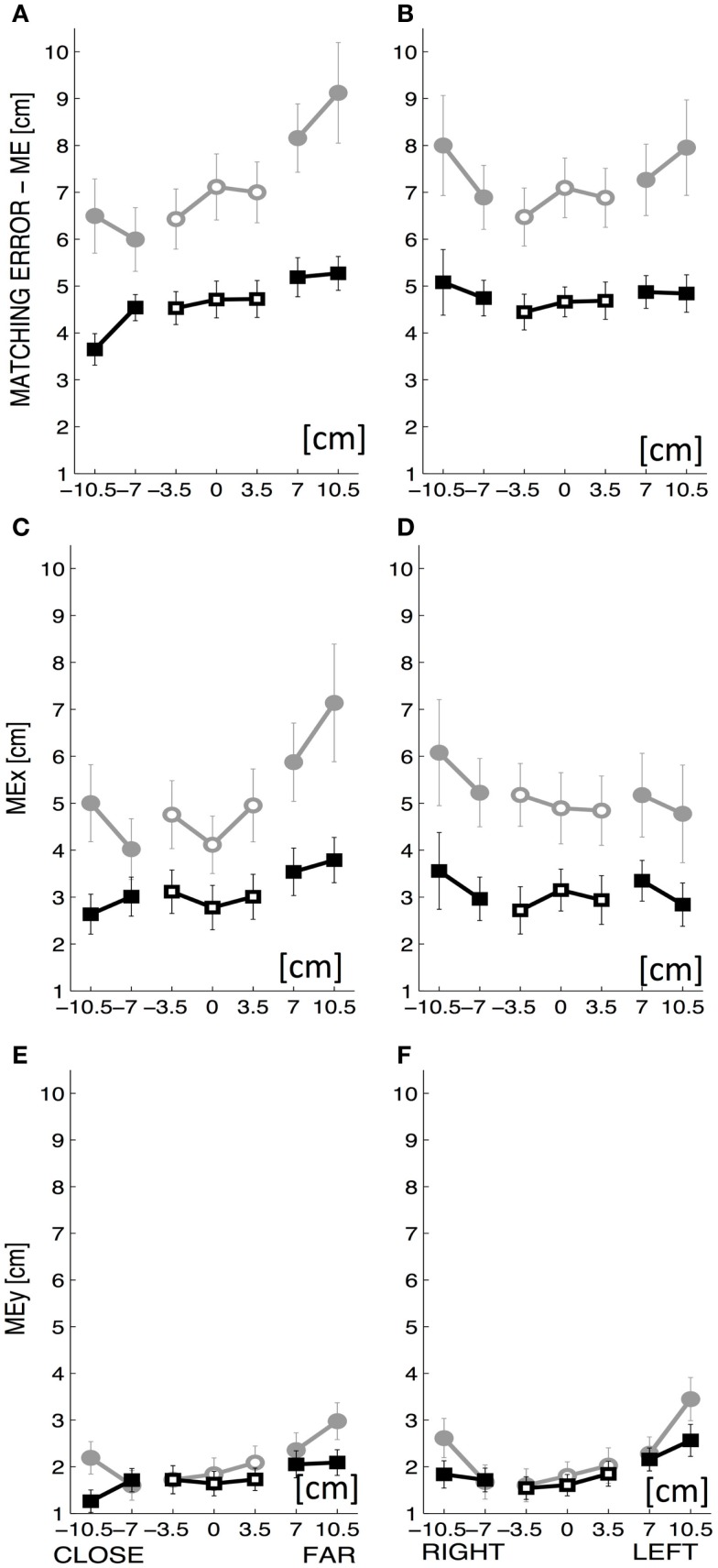
The top row displays the overall matching error (ME: mean ± SE over all subjects) along the *y*-coordinate **(A)** and the *x*-coordinate of the workspace **(B)**. The middle row displays the rightward–leftward component of ME, i.e., ME_*x*_ along the *y*-coordinate **(C)** and the *x*-coordinate **(D)**. The bottom row displays the upward–downward component of ME, i.e., ME_*y*_ along the *y*-coordinate **(E)** and the *x*-coordinate **(F)**. The *x*-coordinate of the workspace goes from left to right, with a range of ±10.5 cm and the *y*-coordinate of the workspace goes from close to far, with a range of ±10.5 cm. Intrinsic-matching test: gray lines; Extrinsic-matching test: black lines. The empty markers correspond to the targets on the midline (0 cm) of the workspace or near to it (±3.5 cm).

To further investigate this dependence on target location, ME was computed separately for the proximal–distal and for the medio-lateral parts of the workspace and we evaluated the difference between the target locations “close vs. far” with respect to the body frontal plane (Figure 4A) and “left vs. right side” with respect to the body midline (Figure 4B). The absolute ME increased significantly with the forward distance from the body (*t* = 5.904, *p* < 0.0001) while it did not change as a function of the lateral displacement.

The ME was also decomposed into two components – ME_*x*_, ME_*y*_ – that represent, respectively, the systematic rightward–leftward shift (ME_*x*_) and upward–downward shift (ME_*y*_) with respect to the target position. In both workspace directions, ME_*x*_ was larger than ME_*y*_ (Figures 4C–F). Both components ME_*x*_ and ME_*y*_ depended on testing conditions [*F*(1,1760) = 174.483 *p* < 0.0001 and *F*(1,1760) = 14.768 *p* < 0.001, respectively] and target locations [*F*(19,1760) = 1.952 *p* = 0.008 and *F*(19,1760) = 2.587 *p* < 0.001, respectively]. ME_*x*_ was also larger when the order of presentation was random [*F*(1,1760) = 27.465 *p* < 0.0001].

Considering the effect of target location in terms of “close vs. far” and “left vs. right side,” while ME_*y*_ was small and presented slight although significant changes along both workspace directions (Figure 4E – along *y*; Figure 4F – along *x*), ME_*x*_ had a relevant increase with the forward distance from the body (*t* = 5.037, *p* < 0.0001) (Figure 4C).

#### Errors along the midline

The matching targets along the midline involve the same arms configuration in both test modalities. However, performance differences between the two testing conditions were consistently observed also for these targets. A three-way ANOVA (factors: matching condition, order of presentation, and distance from the body) for targets located on the midline confirmed better matching performance in terms of absolute ME for the extrinsic-matching test [*F*(1,360) = 42.880, *p* < 0.0001] and for targets closer to the body [*F*(1,360) = 19.711, *p* < 0.0001]. In these “close” targets, the lateral error ME_*x*_, represented the predominant component of the ME. Both ME_*x*_, and ME_*y*_ components showed smaller errors during the extrinsic-matching test [sideways error: *F*(1,360) = 38.368, *p* < 0.0001, under-overshoot error: *F*(1,360) = 6.963, *p* = 0.009] and for targets located closer to the body [*F*(1,360) = 18.354, *p* < 0.0001 and *F*(1,360) = 11.010, *p* = 0.001, for lateral and distal component, respectively].

### Analysis of selected trajectory parameters

**Figure 5:**
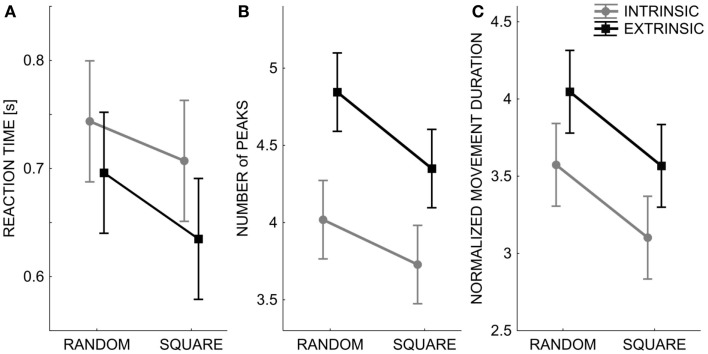
**Spatial and temporal pattern (mean ± SE over all subjects) of the subjects’ movement trajectories: Reaction time (A), smoothness – number of peaks in the speed profile (B), normalized movement duration (C)**. The two lines correspond to the performance in the two different test conditions: Intrinsic-matching test (gray lines) and Extrinsic-matching test (black lines). *X* axis: the two different orders of targets’ presentation: random or sequence of squares.

Although the subjects were not instructed or constrained in any way in how to reach the matched target locations, the temporal and spatial aspects of the reaching trajectories were analyzed in order to detect a possible influence of the two testing modalities. Indeed, differences were consistently found: in the intrinsic modality, the reaction time was significantly higher [*F*(1,1760) = 4.534, *p* = 0.033], but the movement duration was shorter [*F*(1,1760) = 11.684, *p* < 0.001] and the corresponding trajectories were smoother in the sense of having a smaller number of velocity peaks [*F*(1,1760) = 31.301, *p* < 0.0001]. This suggests that the subjects tended to plan shorter and smoother movements in this condition and with fewer feedback corrections (Figure 5). In contrast, in the extrinsic-matching condition the subjects tended to apply more corrections while approaching the intended targets. It is also worth mentioning the comments of some subjects, at the end of the experimental sessions: in the extrinsic-matching test, they reported a “feeling” that the position of the left fingertip guided the motion of the right fingertip.

Moreover, Figure 5 clearly shows that the temporal and spatial features of the matching trajectories were significantly influenced by the modality of target presentation: when the targets were presented in fully random order, the movements were slower [*F*(1,1760) = 11.684, *p* < 0.001] and less smooth [*F*(1,1760) = 9.204, *p* = 0.002] than in the case of square presentation, whereas no significant difference was found for the reaction time.

Finally, the reaction time and the number of peaks of the speed profile significantly changed also according to the target’s location [*F*(19,1760) = 2.882, *p* < 0.0001 and *F*(19,1760) = 1.936, *p* = 0.009]. No significant difference was found in the movement duration.

## Discussion

### Upper limb position sense: Quantitative assessment

In medicine, detecting the presence of dysmetria is an important source of information for a neurologist or even a general practitioner when evaluating a subject. The finger-to-nose test is the typical preliminary semi-quantitative test used for evaluating the position sense. One of the aims of the proposed comparative analysis of the extrinsic vs. intrinsic-matching modality is to extend and reinforce the understanding of the physiological and possibly the pathological correlates of this aspect of sensorimotor integration. The ability to locate one hand or the fingers with respect to the contralateral hand while moving in a common workspace is important in daily life activities, when people interact with the environment by reaching, manipulating, or moving objects. How is the bimanual information combined and used to estimate the position of our limbs in space? Are there specific differences in the use of the bimanual proprioceptive feedback in dependence of the task to be executed? In this study, this issue is tackled by asking subjects to match the position of their left middle fingertip – passively moved in different workspace’s positions – by actively reaching it with the contralateral right fingertip according to two modalities:

– in the same workspace location (extrinsic test).
– in the mirror-symmetric workspace location (intrinsic test).

Generally, the evaluated MEs are comparable with those reported by other studies (Dukelow et al., 2010). However, the novelty of this study is the comparison of the position sense errors under the two test modalities.

Despite the fact that subjects had not a uniform behavior, significant differences were found between the two testing modalities. When subjects had to put both hands in the same location, most of them executed the task with smaller errors, in a more accurate and less variable way – smaller systematic shift and contraction–expansion of the workspace – than when they had to position their hands in mirror-symmetric locations with respect to the body midline. Subjects may focus more on their fingertips in the extrinsic-matching test that explicitly requires to match two-endpoint positions.

This could partially explain the better matching results, since not only subjects seem to represent limb positions in extrinsic coordinates (Kalaska et al., 1990; Prud’homme and Kalaska, 1994; Tillery et al., 1996) but also because, as (Fuentes and Bastian, 2010) suggested, focusing on endpoint coordinates leads to more precise estimate of the matching position.

### Mapping the position sense across the 2D workspace

While several studies investigated the arm position sense in different conditions or focused on the integration of visual and proprioceptive feedback, only few studies (van Beers et al., 1999; Ansems et al., 2006; Bagesteiro et al., 2006; Goble and Brown, 2008; Jones and Henriques, 2010; Schmidt et al., 2013; Henriques et al., 2014) addressed the problem of mapping proprioception across the workspace. Recent studies (Fuentes and Bastian, 2010; Wilson et al., 2010) demonstrated that proprioceptive acuity is not uniform across the workspace: in ipsilateral-matching tasks, Wilson et al. (2010) found that the position error increases for targets that are far from the body and Zuckerman et al. (1999), Rosenbaum and Chaiken (2001), Adamo et al. (2007), and Fuentes and Bastian (2010) showed that the error grows with greater elbow angle excursions. In this work, we found that similar conclusions hold in the proposed bimanual task: the ME appears to increase when moving from targets that are closer to targets that are farther from the body in both testing modalities. The difference between proximal and distal targets may be due to the limb configurations that imply differences in geometry, sensory noise, stretch of the muscles, and limb stability (Fuentes and Bastian, 2010; Wilson et al., 2010). However, error values in the extrinsic-matching test were smaller with respect to the intrinsic-matching test and, interestingly, the subjects’ performance was better in the extrinsic modality also along the midline, where the matching targets were the same for both tests.

This suggests that the matching performance of the subjects could be determined both by raw limb kinematics and proprioceptive information, and by the explicit representation of the task: the goal of reaching for the physical location of the fingertip in space plays a dominant role compared to the more abstract and less functional goal of matching a proprioceptive representation of a body configuration.

### Analysis of the matching movements

The main focus of our task is the final matching results and not how subjects achieved this goal. While we acknowledge that the shape of the trajectory, the movement duration, and the reaction time were free variables in our experiment, we believe that they can be of interest for evaluating to which extent these parameters were dependent on the matching modality. The analysis of the movements suggests that the two testing modalities may be associated with different strategies. The movements in the intrinsic-matching test had slower reaction times but were smoother and faster with respect to the movements in the extrinsic-matching test. This could be due to a variety of factors and it is consistent with the well-known speed–accuracy trade-off principle: as the final matching positions were more accurate, the movement became slower. However, the two strategies are not equivalent and the differences are compatible with the following hypotheses (although they are not the only possible ones): if people represent limb positions in extrinsic coordinates as suggested by some authors (Kalaska et al., 1990; Prud’homme and Kalaska, 1994; Tillery et al., 1996) and tend to apply this same strategy even when the intrinsic-coordinate planning would simplify the task, one would expect an increase in the reaction time. This is because the subjects would need first to compute the position of the left fingertip in the task space and subsequently to flip the target’s coordinates over the right workspace. Then, they would directly move to the estimated target location without the need to adjust their planned movement. Conversely, in the extrinsic task, subjects would move toward their own contralateral fingertip and adjust their movements while approaching it. This would result in less smooth and slower reaching movements (due to longer movement time), but would lead to a better overall matching performance. A possible, although not unique, explanation may be that when the hands become closer to each other, the proprioceptive information coming from the two arms may be combined to give a better estimate of their concurrent spatial location, thus bringing to a more accurate completion of the task.

### Clinical implications

The reported results are relevant to clinical applications at least from two points of view: (i) for the quantitative assessment of position sense while highlighting systematic differences in the outcome measures obtained under the two different testing modalities; (ii) for programing rehabilitative exercises, suggesting to explore the use of the contralateral hand as target for the impaired arm movements.

### Limitation of the study

In the proposed protocol, the matching task in intrinsic coordinates required crossing of the two arms for those targets lying on the contralateral hemi-space with respect to the matching arm. We rarely use our hands in crossover positions and this task requirement may affect proprioception. However, in the reported experiments, the errors were very similar in both hemi-spaces, whether or not matching would require crossing the two arms.

There are other features of the experimental set-up and protocol that may have influenced the results, such as the vertical distance between the planes of action of the two hands, the tactile feedback due to the contact with the holders of the arm and fingertip, as well as the fact that the left reference hand was passively and not actively moved. Moreover, the targets were presented in a sequential order and the matching limb was not repositioned passively to the true target location, because this would alert the subject about the characteristic of his error. This prevented learning, but may have induced error propagation from a target location to the next one, including the positions along the midline.

Finally, matching was always executed by actively moving the dominant hand. The choice of the matching hand could influence the task performance due the asymmetry between the two hands (Adamo and Martin, 2009; Martin and Adamo, 2011; Adamo et al., 2012). For example, the non-dominant hand would require more feedback control than the dominant hand (Bagesteiro and Sainburg, 2002; Wang and Sainburg, 2007; Przybyla et al., 2013), would be more accurate with proprioceptive targets (Goble and Brown, 2008), and would have different sensorimotor gains (Adamo and Martin, 2009; Plaisier and Ernst, 2012; Squeri et al., 2012; Wong et al., 2014). Moreover, the choice of the hand may be also associated to a different attentional bias (Serrien, 2009; Buckingham et al., 2010) and responsibility assignment (White and Diedrichsen, 2010). Therefore, if we change these aspects of the protocol, we can expect differences between the performance of the two hands, with respect to both the final MEs and the spatio-temporal characteristics of the trajectories.

## Conflict of Interest Statement

The authors declare that the research was conducted in the absence of any commercial or financial relationships that could be construed as a potential conflict of interest. The Guest Associate Editor Giovanni Abbruzzese declares that, despite being affiliated to the same institution as authors Riccardo Iandolo, Pietro Morasso, and Maura Casadio, the review process was handled objectively and no conflict of interest exists.
